# Oxidative phosphorylation patterns in pituitary adenoma/neuroendocrine tumors

**DOI:** 10.1007/s11102-026-01658-w

**Published:** 2026-03-11

**Authors:** Maaia Margo Jentus, René G Feichtinger, Willem E Corver, Sara Huber, Laura Ebner, Iris Pelsma, Leontine Bakker, Wouter van Furth, Marco Verstegen, Nienke Biermasz, Johannes A Mayr, Hans Morreau

**Affiliations:** 1 Department of Pathology, Leiden University Medical Center (LUMC), Albinusdreef 2, 2333ZA Leiden, The Netherlands; 2 Centre for Endocrine Tumors Leiden (CETL), Pituitary Center, Leiden University Medical Center (LUMC), Leiden, The Netherlands; 3 University Children’s Hospital, Salzburger Landeskliniken (SALK) and Paracelsus Medical University (PMU), Salzburg, Austria; 4 Department of Biosciences and Medical Biology, Paris Lodron University of Salzburg, Hellbrunnerstrasse 34, Salzburg, 5020 Austria; 5 Department of Medicine, Division of Endocrinology, Leiden University Medical Center, Leiden, The Netherlands; 6 Department of Neurosurgery, Leiden University Medical Center, Leiden, The Netherlands; 7 Research Program for Receptor Biochemistry and Tumor Metabolism, Department of Pediatrics, University Hospital of the Paracelsus Medical University, Salzburg, Austria; 8 Department of Human Genetics, Paracelsus Medical University (PMU), Salzburg, Austria

**Keywords:** Oxidative phosphorylation, Pituitary adenoma, PitNET, Electron transport chain, Cellular respiration

## Abstract

**Purpose:**

Pituitary neuroendocrine tumors (PitNETs), also known as pituitary adenomas, exhibit marked lineage-specific heterogeneity. The underlying molecular biology of certain tumor/adenoma types, particularly gonadotroph tumors/adenomas (SF1-lineage) — which typically exhibit stable genomes — remains poorly understood. This study aimed to define expression patterns of oxidative phosphorylation (OXPHOS) system subunits across PitNET/adenoma lineages.

**Methods:**

Immunohistochemistry was performed in 43 previously molecularly and histologically classified PitNETs/adenomas on tumor and normal adenohypophyseal tissue for VDAC1 (porin) to assess mitochondrial density and the expression of OXPHOS-subunits. Quantified staining intensity scores were used for statistical analyses, and mtDNA sequencing was successful in 21 tumors/adenomas.

**Results:**

Mitochondrial density was significantly increased in PitNETs/adenomas compared with normal tissue. Alterations in OXPHOS subunits expression were non-uniform: complex I deficiency was the most frequent abnormality, often associated with disruptive mtDNA mutations, particularly in genomically stable gonadotroph tumors/adenomas. Two corticotroph tumors/adenomas with near-haploid genomes also harboured disruptive complex I mutations. Alterations in other complexes were less common and typically occurred in combination. Staining heterogeneity was frequent (24/43 tumors/adenomas), including focal expression loss, especially in SF1-lineage and all mtDNA-mutated tumors/adenomas, but also present in tumors/adenomas without mtDNA mutations.

**Conclusions:**

PitNETs/adenomas display lineage-specific and highly heterogeneous OXPHOS-system phenotypes. Complex I deficiency and mtDNA mutations occur not only in genomically stable gonadotroph tumors/adenomas but also in highly disrupted corticotroph tumors/adenomas with a near-haploid genome. Further studies including sequencing of nuclear-encoded OXPHOS-related genes are required to clarify the contribution of altered OXPHOS-subunit expression to PitNET/adenoma biology and potential clinical applications.

**Supplementary Information:**

The online version contains supplementary material available at 10.1007/s11102-026-01658-w.

## Introduction

Pituitary Neuroendocrine Tumors (PitNETs), also known as pituitary adenomas, are increasingly diagnosed, with a prevalence of 78–116 cases per 100 000 people in the last 15 years [1]. While some PitNET/adenoma types mostly treated with medication, surgical resection is performed for approximately a half of clinically diagnosed PitNETs/adenomas. Among those > 40% are non-functional gonadotroph tumors/adenomas (SF1-lineage), ~ 15% are corticotroph tumors/adenomas (TPIT-lineage), and ~ 30% are tumors/adenomas of PIT1-lineage. Recently, we explored chromosomal alteration patterns in diverse PitNET/adenoma subtypes due to relatively low occurrence of somatic mutation hallmarks in sporadic tumors/adenomas, confirming previous observations [2–5]. Tumors/adenomas with aggressive clinical behaviour showed massive chromosomal losses (TPIT-lineage) which are likely to be mutually exclusive with *USP8*-mutations. Tumors/adenomas of PIT1-lineage mostly showed complex patterns of chromosomal losses and gains, with the mechanisms still to be elucidated. Gonadotroph tumors/adenomas (SF1-lineage) do not cause hormone excess and usually present as large tumors/adenomas causing mass effects, often require multiple operations due to difficulty of complete initial surgical resection. These tumors/adenomas mostly show stable genome and no somatic mutations, challenging the search for biomarkers and therapeutical targets. Prior to introduction of systematic staining for transcription factors, a subset of PitNETs/adenomas was classified as pituitary oncocytoma/oncocytic null-cell adenomas (WHO Tumor Classification 2017 and prior), which most likely was represented by gonadotroph tumors/adenomas [6]. In this historical tumor/adenoma type, mtDNA mutations in complex I (CI) of electron transport chain (ETC) were repeatedly reported [7–9]. Kurelac et al. reported a series of 48 pituitary adenomas, in which a correlation was found between CI disruptive mutations, the oncocytic phenotype and low number of chromosomal aberrations [8]. Complex II alterations are known drivers in subset of tumors/adenomas, which occur mostly in the context of SDH-deficient tumor syndrome (hereditary phaeochromocytoma-paraganglioma syndromes), and are less detected sporadically [1, 10, 11]. To our knowledge, no mutations affecting respiratory complexes III-V were reported in pituitary tumors/adenomas.

Abnormal mitochondria and oncocytic phenotype are often described as going hand in hand [12]. It is worth to mention that oncocytic changes also occur in the normal adenohypophysis [13]. Furthermore, there is room for subjective interpretation in the definition of oncocytic phenotype. While oncocytes have big eosinophilic cytoplasm, not all cells with such cytoplasm are true oncocytes. The eosinophilic appearance in some lesions may be attributed to the accumulation of various cellular components, rather than an increased number of deviant morphology of mitochondria [14]. Ultrastructural confirmation by electron microscopy remains the golden standard to demonstrate hyperplasia of abnormal mitochondria [8, 13, 15, 16]. Immunohistochemistry for subunits of the respiratory chain can be utilized to visualize mitochondria [12]. The oncocytic features with corresponding abnormalities in mitochondria are widely described in other endocrine and non-endocrine organs [12, 17, 18]. For PitNETs/adenomas, the situation is likely to be more even complex, as the volume of mitochondria is different among different subtypes in pituitary adenomas; for example, the volume of mitochondria in prolactinomas is larger than those in growth hormone producing tumors/adenomas [19]. Somatotroph tumors/adenomas with oncocytic cells show similar cytokeratin patterns and higher proliferative activity, which is not correlated with local aggressiveness [20]. Acidophilic stem cell tumors/adenomas are per definition oncocytic [1]. Anyways, there is growing consideration of mitochondria as probable therapeutic target in PitNETs/adenomas as mitochondrial alterations have commonly been recognized in these tumors/adenomas [21–23]. The process of oxidative phosphorylation within mitochondria represent the main source of energy (adenosine triphosphate, ATP) under aerobic conditions in eukaryotic cells [24]. The core elements are ETC, composed of complexes I-IV and ATPase, often called complex V. The complexes consist of diverse amount of subunits and organized in supercomplexes, embedded in the inner mitochondrial membrane [25]. Prone to mutations mitochondrial DNA (mtDNA) codes only for 13 proteins all of which are subunits of OXPHOS-complexes. Most of the subunits of the complexes are nuclear-encoded and are imported into mitochondria where large multi-subunit complexes are assembled. Beside those, the whole orchestra of supporting proteins and signalling systems are necessary for proper function of cellular respiration. Beyond the roles in energy production, participation in cell signalling, regulation of apoptosis, mitochondria continuously gain attention in the context of tumorigenesis.

In this study, we explored mitochondrial density, mtDNA mutation status and immunohistochemical expression of ETC- and ATPase subunits in a previously molecularly defined and subtyped PitNET/adenoma cohort [2].

## Materials and methods

### Sample collection

Material from 43 sporadic PitNETs/adenomas previously described by our group was available for additional analyses, with case numbering consistent the earlier study [2]. Adjacent normal adenohypophyseal tissue (with preserved organoid architecture and appropriate expression of lineage-specific transcription factors and hormones) was available in nine cases. All analyses were covered by the ethical approval of the original study (Medical Ethics Review Committee Leiden, G19.011).

## Clinical and pathological characteristics of included cases

The present cohort comprised 25 female (58%) and 18 male (42%) patients, with a mean age of 46.1 years (median 48, range 19–79). Female patients (mean 41.9 years, median 31, range 19–74) were averagely younger than male patients (mean 51.9 years, median 49.5, range 28–79).

Tumors/adenomas of the PIT1-lineage represented the largest group (*n* = 20), followed by SF1-lineage (*n* = 13), TPIT-lineage (*n* = 9), and one multilineage tumor/adenoma of PIT1/SF1-lineages.

All PIT1-lineage tumors/adenomas were clinically functional. Among TPIT-lineage tumors/adenomas, five were clinically functioning and four were silent corticotroph tumors/adenomas. Of the 13 SF1-lineage tumors/adenomas, 12 were clinically non-functioning and one was reported to express FSH.

Twenty-five of 43 tumors were invasive, and 18 were non-invasive respectively. Aggressive pituitary tumors (APT) were defined according to the European Society of Endocrinology (ESE) criteria [26].

## Assessment of oncocytic phenotype

Haematoxylin and eosin (H&E)-stained sections were independently evaluated for oncocytic features by two pathologists (MJ and HM). Tumors/adenomas were categorized as non-oncocytic; oncocytic-metaplasia like, or oncocytic.

Tumors were classified as oncocytic when ≥ 70% of tumor cells displayed the characteristic cytoplasmic phenotype. Cases showing focal oncocytic change (< 70%) or not fully developed cytoplasmatic features were classified as oncocytic metaplasia-like. Tumors lacking oncocytic features were designated non-oncocytic.

In cases of discrepant scoring, consensus was reached by joint review.

Interobserver agreement between the two pathologists for the three-tier oncocytic classification was fair (Cohen’s κ = 0.31, *p* < 0.005). When simplified to binary classification, agreement improved to moderate (κ = 0.54, *p* < 0.001) when “metaplasia-like change” was grouped with “oncocytic,” and remained fair (κ = 0.32, *p* = 0.042) when grouped with “non-oncocytic.” Because oncocytic morphology on H&E does not directly quantify mitochondrial content and showed only limited interobserver reproducibility in our dataset, we therefore relied on VDAC1 immunohistochemistry as an objective surrogate of mitochondrial mass (discussed below).

## Immunohistochemistry for mitochondrial density and subunits of OXPHOS-system

### Materials and considerations

Immunohistochemistry was performed for subunits of the CI-CV and porin (VDAC1) as described previously [7]. VDAC1 staining was used to assess mitochondrial density and potential alterations in mitochondrial biogenesis within PitNETs/adenomas, both intratumorally and compared to normal adenohypophyseal tissue. Porin is located in the outer mitochondrial membrane and is frequently used as a marker for mitochondrial mass [27–29].

The following primary antibodies were used:

- VDAC1 (porin): mouse anti-VDAC1 (Abcam, ab14734, RRID: AB_443084; 1:1000, 1 h).
- Complex I: rabbit anti-NDUFB8 (Abcam, ab192878, RRID: AB_2847808; 1:500, 1 h).
- Complex II: mouse anti-SDHA (Abcam, ab14715, RRID: AB_301433; 1:2000, 1 h).
- Complex III: mouse anti-UQCRC2 (Abcam, ab14745, RRID: AB_2213640; 1:1000, 1 h).
- Complex IV: mouse anti-MT-CO1 (Abcam, ab14705, RRID: AB_2084810; 1:1000, 1 h).
- Complex V: mouse anti-ATP5F1A (Abcam, ab14748, RRID: AB_301447; 1:2000, 1 h).

### Scoring

Scoring and statistical analyses were performed as previously described [29, 30]. Immunohistochemical expression was semi-quantitatively assessed using a four-tier intensity scale (0 = no staining, 1 = weak, 2 = moderate, 3 = strong), allowing half-point increments.

For each staining, a composite score was calculated by multiplying the staining intensity by the percentage of tumor area exhibiting that intensity.

In tumors without visible heterogeneity, the entire tumor area (100%) was scored as a single region.

When heterogeneous staining was observed, morphologically distinct regions (minimum size 5% of tumor area) demonstrating clearly decreased or increased expression were scored separately. These regional scores were weighted according to the percentage of tumor surface involved and summed to obtain the final tumor score.

Immunohistochemical expression levels in tumor/adenoma tissue were compared with adjacent pre-existing adenohypophyseal tissue when available. Otherwise, mean expression levels derived from normal adenohypophyseal tissue across cases were used as reference.

Quantification was performed independently by two observers (MJ, HM), and the mean interobserver score was used for all analyses. Interobserver agreement as assessed using a two-way random-effects model with absolute agreement (intraclass correlation coefficient) showed good to excellent interobserver reliability across all stainings.

## Assessment of heterogeneity

Tumors/adenomas frequently showed heterogeneous or patchy staining patterns and focal loss of expression. To semi-quantify intratumoral heterogeneity, variability in staining intensity was first assessed in normal adenohypophyseal tissue by calculating the 95% confidence interval (CI) of the mean score for each marker. Tumors/adenomas were classified as *heterogenous* for a given staining when the intratumoral range of staining scores (maximum minus the minimum observed regional score) exceeded the width of the 95% CI derived from normal adenohypophyseal tissue.

## Definition of altered expression

Increased or decreased immunohistochemical expression in tumor/adenoma tissue was defined as a relative change of 25%, 50%, or 75% in the composite score value compared with the mean score of normal adenohypophyseal tissue or the corresponding adjacent normal adenohypophyseal tissue when available.

**Fig. 1:**
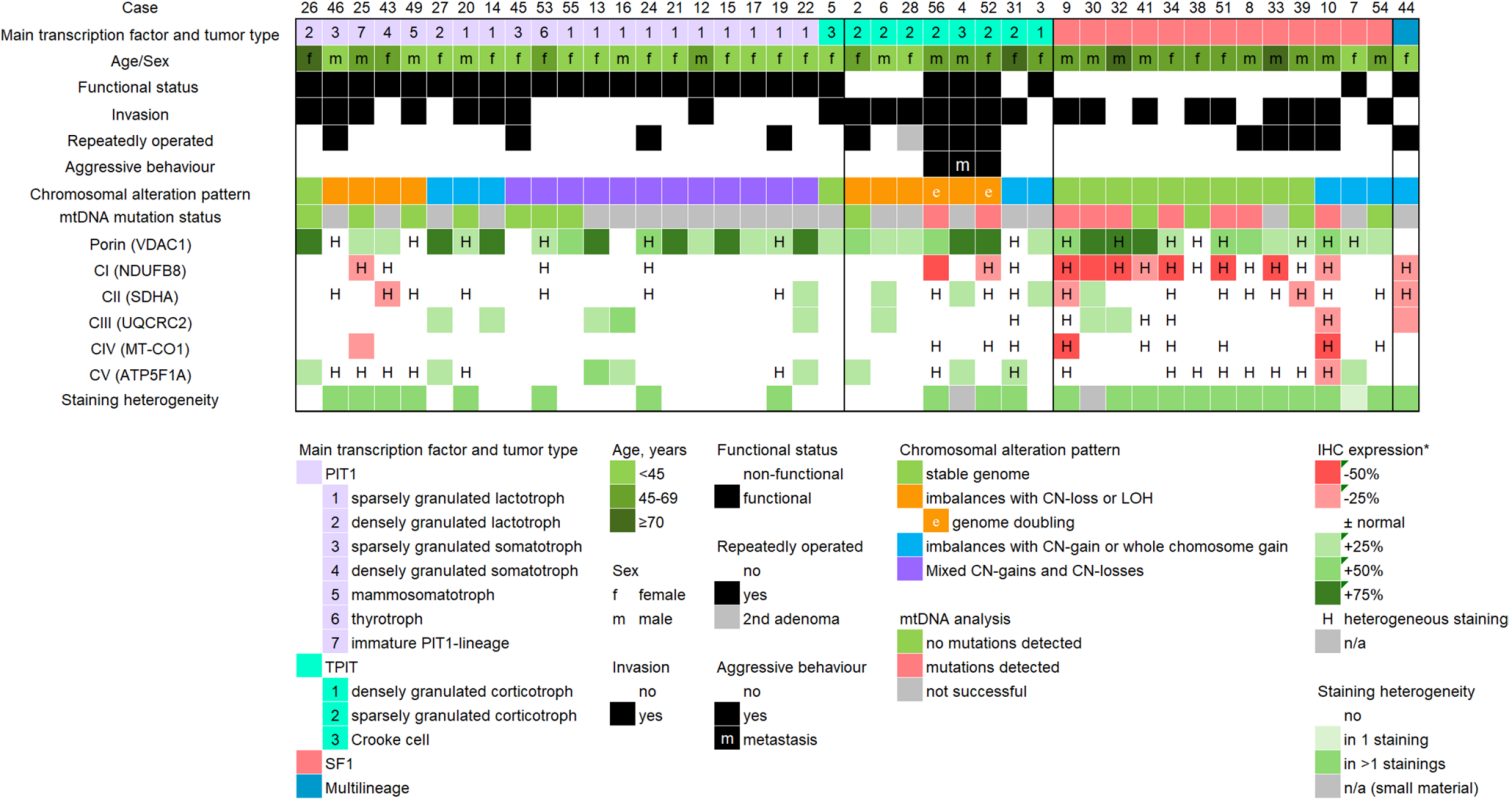
Overview of the study cohort. Selected clinical characteristics of the patients, PitNET/adenoma subtypes (grouped by lineage), chromosomal alteration patterns, immunohistochemistry for porin (VDAC1) and subunits of complexes I-V, and mtDNA mutation status (where available) are shown. Heterogeneous/patchy immunohistochemical staining was observed across all lineages and stainings. No mtDNA mutations were observed in tumors/adenomas of PIT1-lineage. In contrast, the majority of SF1-lineage tumors/adenomas harboured disruptive mtDNA mutations in complex I, which were associated with reduced immunohistochemical expression of NDUFB8 (complex I subunit) *in %, compared to normal adenohypophyseal tissue

In tumors/adenomas harbouring mtDNA mutations affecting complex I and displaying heterogeneous staining, regions with reduced NDUFB8-expression approximately corresponding to the heteroplasmy percentage of the mutation were identified. For these cases, expression levels of mutation-associated regions (rather than the mean score of the entire tumor) was utilized for analyses and Fig. 1.

Additionally, intratumoral ratios were calculated by comparing mutation-associated regions with adjacent lesional regions showing preserved expression. These intratumoral ratios were exclusively used for visualization of regional differences in Fig. 3B.

### Additional molecular studies

#### DNA extraction

Fresh-frozen material was available for 21 samples, from which DNA was isolated using ten 20-µm cryosections per case.

### mtDNA sequencing

The detailed protocols are described in the Supplementary Methods.

MtDNA was amplified from proteinase K-digested fresh-frozen tumor/adenoma tissue using long-range PCR.

Long-read mtDNA sequencing library was prepared using the Oxford Nanopore Technologies Native Barcoding Kit (SQK-NBD114.24).

Sequencing was performed on a P2 Solo sequencing device using FLO-PRO114M flow cells. Basecalling, demultiplexing, and alignment against human reference genome (GRCh38) were performed in real time using MinKNOW (v.24.02.6). BAM files were sorted and indexed using SAMtools (v.1.18) [31], and variant calling was performed with freebayes (v.1.3.6) [32].

### Evaluation of pathogenicity of detected mtDNA variants

The pathogenicity of detected mtDNA variants was evaluated based on population frequency and existing annotations in the MitoMap database (https://www.mitomap.org), which integrates data from gnomAD, GenBank, and Helix. MitoMap annotations were used to identify previously reported variants and their known or suggested pathogenic relevance.

For novel mtDNA variants, in silico pathogenicity prediction was performed using multiple tools, including Apogee2, Hmtvar, AlphaMissense, BayesDel_addAF, DEOGEN2, LIST_S2, MutationAssessor, PhyloP100, PROVEAN, Sift4G, GERP RS, and Varity_R.

### Statistical methods

All statistical analyses were performed using GraphPad Prism version 10.2.3 (GraphPad Software, USA) and IBM SPSS Statistics 29.0.0.0 (241). Given the limited sample size, the study was exploratory in nature and primarily relied on descriptive statistics, with inferential analyses interpreted cautiously. Normality of staining intensity distributions in grouped samples was assessed using the Shapiro–Wilk test. For comparisons between groups, Welch’s t-test was applied when data approximated a normal distribution, whereas the Mann–Whitney U test was used for non-normally distributed data. Associations between VDAC1 expression, oncocytic phenotype, mtDNA mutation status, and clinical parameters were explored using Spearman rank correlation, simple linear regression, and logistic regression, as appropriate. All tests were two-sided, and p values < 0.05 were considered statistically significant.

## Results

An overview of the study cohort is presented in Fig. 1, including clinical characteristics, immunohistochemistry and summarized molecular data. Immunohistochemistry for VDAC1 (to assess mitochondrial density) and OXPHOS-subunits was successful on 43 tumors/adenomas, comprising 20 PIT1-lineage, 13 SF1 lineage, 9 TPIT-lineage, and one multilineage tumor/adenoma (SF1/PIT1-lineages). Additional mtDNA analysis was successful in 21 cases, of which nine harboured mtDNA mutations (Table 1).

### Mitochondrial density

Mitochondrial density, assessed by porin (VDAC1) expression, was significantly higher in tumor/adenoma tissue (162.3 ± 29.2) compared with normal adenohypophyseal tissue (111.1 ± 22.1; *p* < 0.0001). Thirty-eight of 43 tumors/adenomas (88.4%) showed mean intratumoral VDAC1 staining intensity above the normal range.

### Heterogenous/patchy staining

**Fig. 2:**
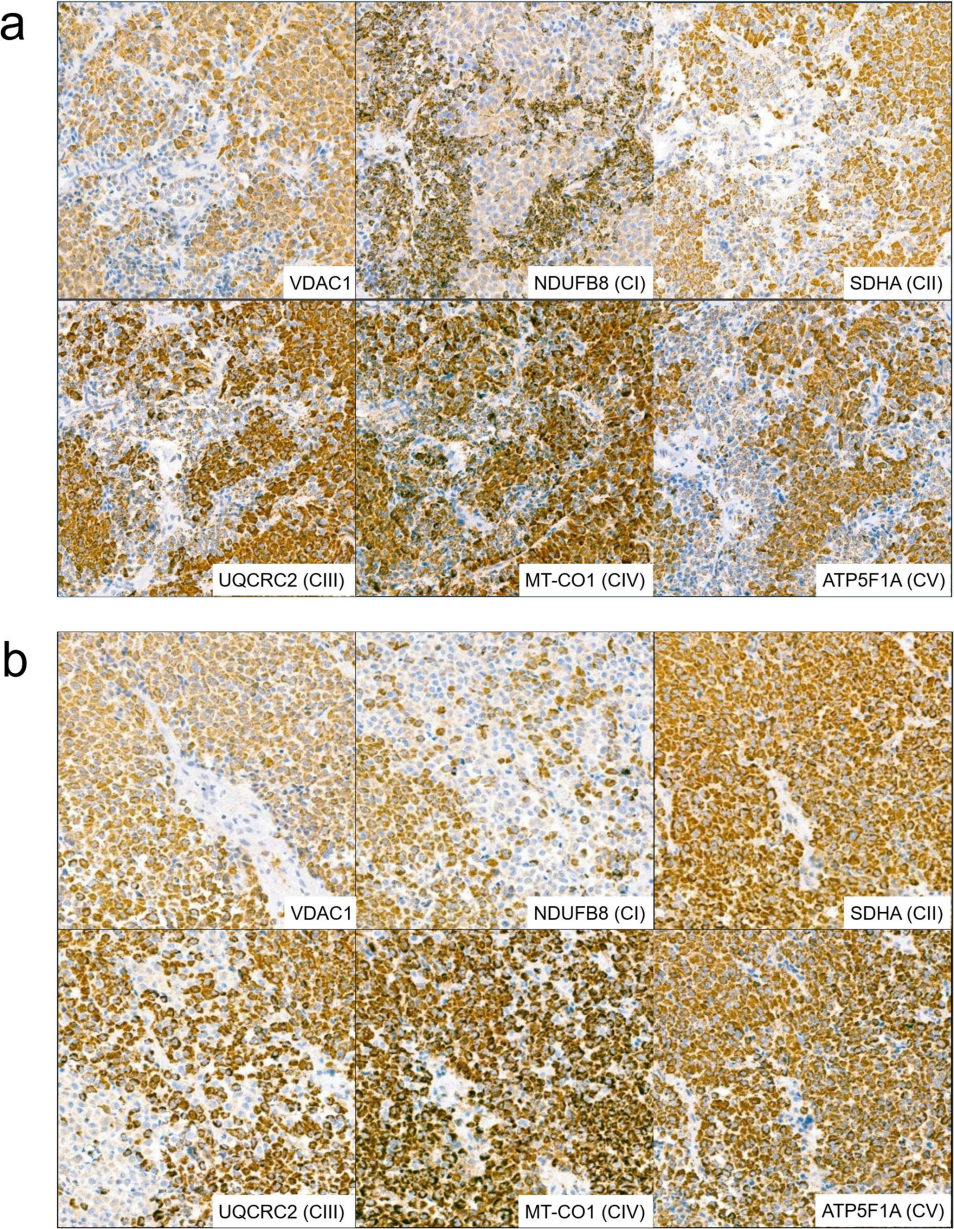
Examples of heterogenous/patchy intratumoral staining (**a**) Case 34 (**b**) (SF1-lineage), m.11038del (91%) detected (**c**) Case 41 (**d**) (SF1-lineage), no mtDNA mutations detected. Magnification x10

Of 43 tumors/adenomas, 24 showed heterogenous/patchy staining pattern (Fig. 2), whereas 17 showed homogenous staining. Two samples were too small to assess heterogeneity (1xSF1 and 1xTPIT-lineage).

Among 24 tumors/adenomas with heterogeneous staining, 23 were patchy in 2 or more stainings, and four tumors/adenomas demonstrated a heterogeneous pattern in all six stainings. Heterogenous staining was most frequently observed for SDHA (CII, 19/24), followed by NDUFB8 (CI, 17/24), ATP5F1A (CV, 16/24), VDAC1 (porin, 15/24), MT-CO1 (CIV, 9/24), and UQCRC2 (CIII, 5/24).

Staining heterogeneity was most prevalent in SF1-lineage tumors/adenomas (11/12). In contrast, approximately only half of TPIT-lineage tumors/adenomas (3/8) and PIT1-lineage tumors/adenomas (11/23) showed heterogeneous staining. Moreover, tumors/adenomas of PIT1-lineage did not show heterogeneity in UQCRC2 (CIII) and MT-CO1 (CIV) stainings.

### Immunohistochemical expression of CI-CV subunits

An overview of immunohistochemical staining intensities per case is provided in Fig. 1 and summarized by lineage in Table 1.

**Table 1:** Summary of immunohistochemical staining patterns per lineage

	SF1 (*n* = 13)	PIT1 (*n* = 20)	TPIT (*n* = 9)	Multilineage (*n* = 1)
NDUFB8 (CI)				
Increased	0	0	0	0
Decreased	8	1	2	1
± normal	5	19	7	0
SDHA (CII)				
Increased	1	1	3	0
Decreased	2	1	0	1
± normal	10	18	6	0
UQCRC2 (CIII)				
Increased	2	5	1	0
Decreased	1	0	0	1
± normal	10	15	8	0
MT-CO1 (CIV)				
Increased	0	0	0	0
Decreased	2	1	0	0
± normal	11	19	9	1
ATP5F1A (CV)				
Increased	1	5	3	0
Decreased	1	0	0	0
± normal	11	15	6	1

Decreased NDUFB8 (CI) expression was observed most frequently (*n* = 12), occurring either in isolation (*n* = 8) or in combination with decreased expression of other complexes (4). Decreased NDUFB8-expression was predominantly observed in tumors/adenomas harbouring mtDNA mutations (*n* = 8) but was also observed in two tumors/adenomas without detectable mtDNA mutations (e.g., Fig. 2b).

Decreased UQCRC2 (CIII) and MT-CO1 (CIV) occurred exclusively in combination with decreased NDUFB8-expression. Decreased ATP5F1A (CV) expression was observed in a single tumor/adenoma and was also a part of a multi-complex deficiency.

Increased expression of NDUFB8 or MT-CO1 was not observed.

In multiple linear regression analysis, SDHA (*p* = 0.004) and ATP5F1A expression (*p* = 0.03) were independently associated with higher VDAC1 expression, reflecting increased mitochondrial density. In contrast, NDUFB8 (*p* = 0.02) and MT-CO1 (*p* = 0.02) showed inverse associations.

**Fig. 3:**
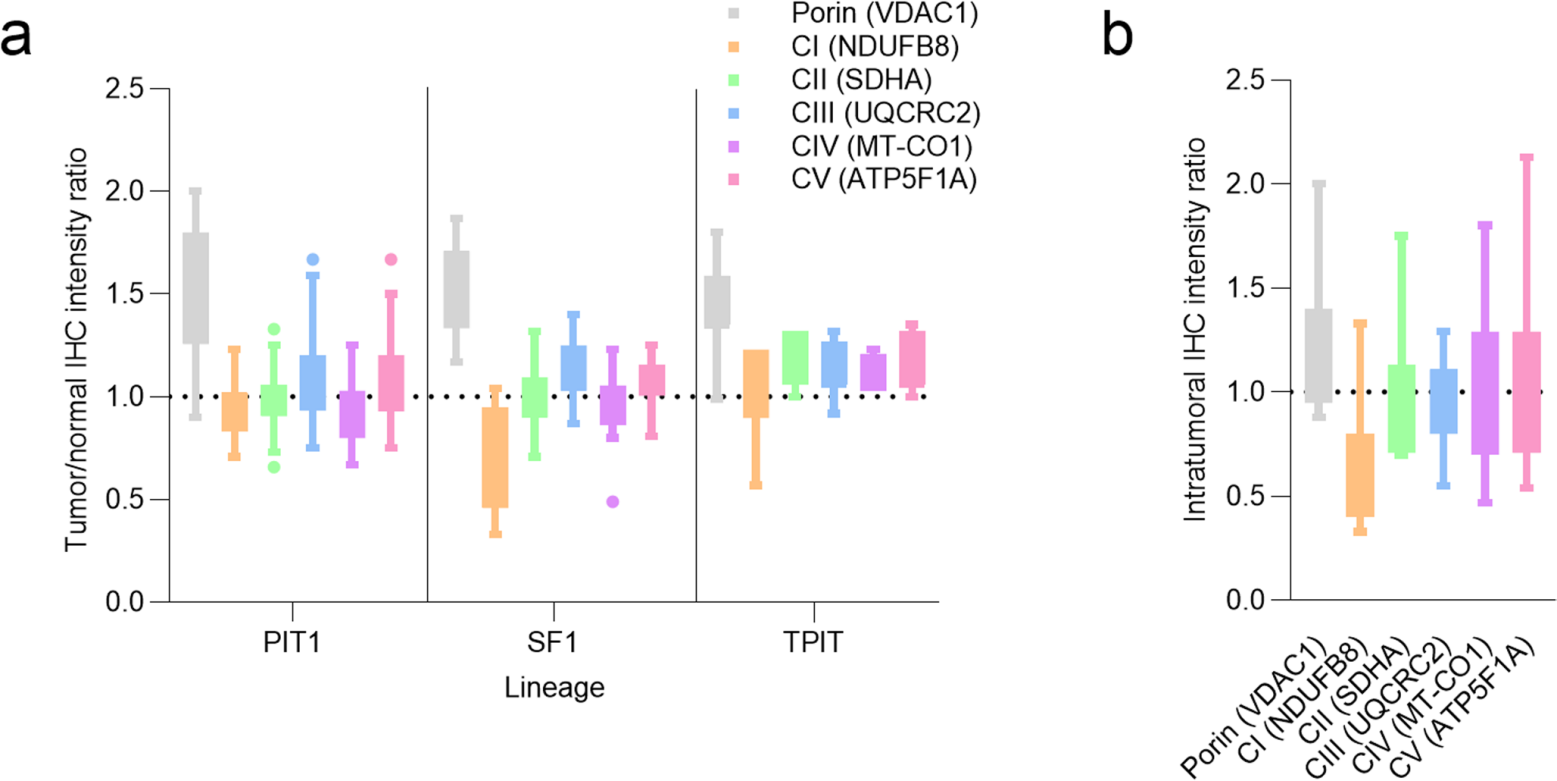
Immunohistochemical staining intensity ratios for subunits of respiratory complexes I–V and VDAC1 in pituitary adenomas/PitNETs (**a**)Tumor-to-normal staining intensity ratios stratified by lineage. The dashed line at 1.0 indicates parity with normal tissue. A lineage-specific trend toward reduced NDUFB8 expression (CI) is observed in SF1-lineage tumors/adenomas, whereas VDAC1 expression, reflecting mitochondrial density, is increased across all lineages (**b**) Intratumoral staining heterogeneity in seven tumors/adenomas harbouring mtDNA mutations affecting complex I. Ratios represent staining intensity in regions corresponding to the heteroplasmic mutation relative to other regions within the same tumor/adenoma

Lineage-specific expression patterns and the overall distribution of immunohistochemical staining intensities are illustrated in Fig. 3.

### mtDNA sequencing

mtDNA mutations were detected in nine of 21 tumors/adenomas in which mtDNA sequencing was successful (Table 1). Two novel missense and one novel loss-of-function variant were identified.

The MT-ND1 m.3631T > C variant results in substitution of a highly conserved serine residue within a transmembrane domain (p.Ser109Pro), spanning amino acid residues 100–120. The variant was absent from gnomAD 4.1 and MitoMap. According to ACMG criteria, it was classified as a variant of uncertain significance (PM2 moderate, PP3 supporting). Multiple in silico prediction tools support a pathogenic effect (Apogee2, Pathogenic, 0.87; Hmtvar, Pathogenic, 0.84; AlphaMissense, Pathogenic, 0.90; BayesDel_addAF; Uncertain; 0.057, T; DEOGEN2, Benign, 0.23, T; LIST_S2, Uncertain, 0.95, D; MutationAssessor, Pathogenic, 4.3, H; PhyloP100, 4.8; PROVEAN, Pathogenic, -4.6, D; Sift4G, Pathogenic, 0.0010, D; GERP RS, 4.5; Varity_R, 0.95).

The MT-ND4 m.11484G > A variant was detected in one tumor/adenoma and affects a highly conserved glycine residue within a transmembrane domain (p.Gly242Asp), spanning residues 224–244. This variant has been reported at relatively low heteroplasmy (25%) in one individual in gnomAD but was absent in gnomAD 4.1 and MitoMap. In the tumor/adenoma sample, heteroplasmy reached 84%. According to ACMG criteria, this variant was also classified as of uncertain of uncertain significance (PM2 moderate, PP3 supporting), with the majority of in silico tools (7/11) predicting a damaging or pathogenic effect (Apogee2, Pathogenic, 0.74; Hmtvar, Pathogenic, 0.88; AlphaMissense, Pathogenic, 1.0; BayesDel_addAF, Benign, -0.19, T; DEOGEN2, Uncertain, 0.44, T; LIST_S2, Uncertain, 0.93, D; MutationAssessor; Pathogenic, 5.2, H; PhyloP100, 9.4; PROVEAN, Pathogenic, -6.0, D; Sift, Pathogenic, 0.0, D; Sift4G, Pathogenic, 0.0, D; GERP RS, 5.1; Varity_R, 0.97).

The novel loss-of-function variant in MT-ND2 (m.5366_5367del; c.896_897del) causes a frameshift leading to a premature stop codon (p.Ser299TyrfsTer10). The variant was absent from gnomAD and MitoMap.

The remaining pathogenic mtDNA variants identified in this cohort had been previously reported (Table 1). An (Table 2) overview of all 161 detected mtDNA variants across the 21 PitNETs/adenomas is provided in Supplemental Material.

**Table 2:** All detected mtDNA mutations

Case (Lineage)	mtDNA variant	Mutant load (%)	Effect	Affected gene(s)	Complex	Reference/prior report
8 (SF1)	m.10158T > C	46	p.Ser34Pro	MT-ND3	C I	Pathogenic in neurological disease [33, 34]
9 (SF1)	m.4412G > A	38	tRNA DHU stem	MT-TM	C I, C III, C IV, C V	Pathogenic in neurological disease [35, 36]
10 (SF1)	m.12923G > A	31	Stop	MT-ND5	C I	Likely pathogenic in neurological disease [37]
30 (SF1)	m.11484G > A	84	p.Gly242Asp	MT-ND4	C I	https://reg.genome.network/redmine/projects/registry/genboree_registry/alleles?refseq=NC_012920.1&begin=11481&end=11581&skip=0&limit=50
32 (SF1)	m.4436_16463del	41	Deletion	MT-ND1, MT-ND2, MT-ND3, MT-ND4L, MT-ND4, MT-ND5, MT-CYB, MT-CO1, MT-CO2, MT-CO3, MT-ATP6, MT-ATP8	C I, C III, C IV, C V	Large deletions reported in human cancers [38, 39].
	m.4974G > A	36	Stop	MT-ND2	CI	Variant of uncertain significance [40] or pathogenic [41]
34 (SF1)	m.11038del	91	Frameshift	MT-ND4	CI	Kidney oncocytoma [42], oncocytic thyroid carcinoma [43], adrenal oncocytoma [7]
51 (SF1)	m.12425del	90	Frameshift	MT-ND5	CI	IHC-negative oncocytic pituitary adenoma [8], nasopharyngeal oncocytic tumor [44]
52 (TPIT)	m.3631T > C	23	p.Ser109Pro	MT-ND1	CI	dbSNP: rs1603219053
56 (TPIT)	m.5366_5367del	42	Frameshift	MT-ND2	CI	This study

All tumors/adenomas carrying mtDNA mutations showed decreased regional NDUFB8-expression accompanied by a complementary increase in mitochondrial density (Supplemental Fig. 1). Intratumoral expression variability in tumors/adenomas harbouring mtDNA mutations is visualised in Fig. 3B.

### mtDNA mutation status and chromosomal alterations

Among the nine tumors/adenomas harbouring mtDNA mutations, six (all SF1-lineage) showed stable genomes without chromosomal alterations. The remaining three showed disputed genomes: two (TPIT-lineage) showed near-haploid genomes with loss of heterozygosity (LOH) affecting multiple chromosomes due to whole chromosome loss with expected or detected endoreduplication, and one (SF1-lineage) showed chromosomal imbalances due to copy number (CN) gain.

No mtDNA mutations were detected in 12 tumors/adenomas. Among these, four exhibited stable genomes (1xPIT1-lineage, 3xSF1-lineage), while eight demonstrated disrupted genomes. Of the latter, three showed imbalances due to CN loss or LOH (2xPIT1-lineage, 1xTPIT-lineage), two showed imbalances due to CN gain (1xPIT1-lienage, 1xSF1-lineage), and three (3xPIT1-lineage) demonstrated mixed pattern with combined CN gains and losses.

### Correlations of clinical parameters with mtDNA mutation status and mitochondrial protein expression

No significant associations were observed between mtDNA mutation status and clinical parameters. A non-significant trend toward higher mutation frequency with increasing age was noted (OR 1.04 per year, *p* = 0.2). mtDNA mutation status was not associated with sex (*p* = 0.66), invasiveness (*p* = 0.61), functional status (*p* = 0.18), or repeated surgery (*p* = 0.40).

In multivariable logistic regression analysis including mitochondrial density (VDAC1) and complex I (NDUFB8) expression, lower CI expression was independently associated with the presence of mtDNA mutations (*p* = 0.03), whereas VDAC1 expression was not (*p* = 0.36). Consistently, CI expression was significantly lower in mtDNA-mutated tumors in univariate analysis (*p* = 0.003). A trend toward increased mitochondrial density in mtDNA-mutated tumors was observed but did not reach statistical significance (*p* = 0.067).

Neither VDAC1 nor NDUFB-expression was significantly associated with invasiveness (*p* = 0.50 and *p* = 0.11, respectively) or repeated surgery (*p* = 0.44 and *p* = 0.47, respectively). NDUFB8-expression showed a weak association with sex (higher expression slightly reduced odds of being male, *p* = 0.028), and a modest positive association with functional status (*p* = 0.40, OR = 1.01 per unit increase), although the overall regression model for functional status did not reach statistical significance.

#### Oncocytic phenotype

Oncocytic morphology was assessed on H&E sections using a three-tier classification. Interobserver agreement was limited (see Methods), and discrepant cases were resolved by consensus.

No significant association was observed between oncocytic morphology and mitochondrial density as assessed by VDAC1 expression. Lineage-specific analyses likewise revealed no consistent association.

Similarly, mtDNA mutation status was not associated with oncocytic morphology or mitochondrial density.

Genome stability was also not associated with oncocytic morphology or mitochondrial density.

Representative cases are shown in Supplemental Fig. 2 to illustrate the lack of concordance between oncocytic appearance and VDAC1 staining intensity. Both morphologically non-oncocytic and oncocytic tumors demonstrated variable mitochondrial density, indicating that routine H&E-defined oncocytic features are not a reliable surrogate for mitochondrial mass in this cohort.

## Discussion

Previously, we explored chromosomal alteration patterns across all lineages of pituitary neuroendocrine tumors (PitNETs)/adenomas [2]. Gonadotroph tumors/adenomas (SF1-lineage) – typically occurring in older patients and presenting as larger, often invasive lesions – showed almost no chromosomal alterations and were characterized by a stable genome or low frequent chromosomal imbalances due to copy-number gains, suggesting distinct biological characteristics from other PitNET/adenoma-lineages.

Kurelac at al. investigated genomic stability in pituitary adenomas with oncocytic phenotype (formerly termed pituitary oncocytoma/oncocytic adenoma) and reported stable genome or copy-number gains in tumors/adenomas together with frequent disruptive mtDNA mutations affecting respiratory complex I [8]. However, following the revision of PitNET/adenoma nomenclature [1] with introduction of transcription factors, the entity of “pituitary oncocytoma” was abandoned. Reassessment of historical cases therefore allows only limited extrapolation to current lineage definitions. These earlier findings motivated us to investigate subunits of the OXPHOS-system, mtDNA mutations and oncocytic features within a molecularly defined and contemporary subtyped PitNET/adenoma cohort.

PitNETs/adenomas commonly show increased mitochondrial density, reflected by elevated VDAC1-expression, compared to normal adenohypophyseal tissue.

Complex I deficiency was the most frequent abnormality and was significantly associated with mtDNA mutations. In multivariable analysis, reduced NDUFB8 expression remained independently associated with the presence of mtDNA mutations, whereas VDAC1 expression did not. Increased SDHA (subunit of CII) and ATP5F1A (subunit of CV) expression was positively associated with mitochondrial mass (VDAC1), whereas NDUFB8 (subunit of CI) and MT-CO1 (subunit of CIV) correlated inversely. This pattern suggests that mitochondrial expansion may reflect compensatory metabolic adaptation, with induction of complexes II and V in the context of impaired or depleted complexes I and IV.

Deficiencies of complexes II–V were uncommon and generally occurred in combination with complex I reduction. Reduced MT-CO1 expression was rare and consistently accompanied complex I deficiency.

A notable observation of current study was the striking intratumoral heterogeneity of ETC-subunit expression. To our knowledge, this is the first detailed description of extensive intratumoral heterogeneity of ETC-subunits expression in PitNETs/adenomas. Such heterogeneous staining — previously described in papillary thyroid carcinoma and non-neoplastic tissue [28, 45] — was common in our cohort, particularly in SF1-lineage tumors/adenomas, and was present in all tumors/adenomas harbouring mtDNA mutations. Similar heterogeneity and/or reduced expression was also observed in multiple tumors/adenomas without detectable mtDNA mutations, suggesting a potential contribution of nuclear-encoded ETC-related genes (not assessed). The coexistence of strongly and weakly stained tumor/adenomas regions supports clonal metabolic divergence. Whether this heterogeneity affects hormone secretion, proliferation, or treatment response in PitNETs/adenomas remains to be determined.

Decreased NDUFB8 (CI) expression and mtDNA mutations were observed predominantly in SF1-lineage tumors/adenomas, but also in two TPIT-lineage tumors/adenomas, characterized by highly unstable near-haploid genomes with extensive loss of heterozygosity. This demonstrates that mitochondrial dysfunction is not restricted to genomically stable tumors. Notably, no mtDNA mutations were detected in PIT1-lineage tumors in our cohort, although this may partially reflect sampling and material availability.

Kurelac et al. reported disruptive CI mtDNA mutation (m.11832G > A) in an oncocytic tumor/adenoma with TSH/PRL-expression (likely PIT1-lineage under current classification) accompanied by copy-number losses along chromosome 13 [8]. Another oncocytic tumor/adenoma without hormone expression harboured a different disruptive complex I mtDNA mutation (m.11873insC), also co-occurring with copy-number losses along chromosome 13. A third oncocytic growth hormone-expressing tumor/adenoma (again, likely PIT1-lineage) showed copy-number losses across chromosomes 10, 13, and 16, harboured a *GNAS* mutation (c.2530 C > T; p.Arg844Cys), and lacked mtDNA mutations. Together, these cases illustrate that the relationship between oncocytic morphology, genome stability, and mitochondrial mutations is not straightforward.

Oncocytic morphology has historically been associated with mitochondrial hyperplasia and mtDNA mutations. However, our findings do not support a robust relationship between oncocytic appearance on H&E and mitochondrial mass, mutation status or genomic stability. Interobserver agreement for oncocytic phenotype in our cohort was only fair, underscoring the limited reproducibility of this feature. Light microscopic assessment is inherently subjective and may be influenced by staining characteristics and tissue processing. Electron microscopy remains the gold standard method for demonstrating oncocytic phenotype but was not performed in this study [8, 13, 15, 16].

Importantly, mtDNA mutation status was not associated with age, sex, invasiveness, functional status, or repeated surgery in our cohort. Although mutations tended to occur in older patients, this did not reach statistical significance when age was analysed as a continuous variable. Similarly, mitochondrial density and complex I expression were not consistently associated with clinical parameters. A modest association between complex I expression and functional status was observed, but the overall regression model did not reach statistical significance, and this finding should therefore be interpreted cautiously.

In summary, mtDNA mutations leading to impaired respiratory complex I expression are observed both, in genomically stable non-functional pituitary tumors/adenomas and functional tumors with near-haploid genomes. Decreased complex I expression is observed also in the absence of mtDNA mutations, suggesting the possible role of nuclear-encoded genes contributing to respiratory chain defects. These finding highlight the biological complexity of PitNETs/adenomas and suggest that mitochondrial dysfunction represents an additional layer of heterogeneity beyond lineages and chromosomal alteration patterns. Larger studies integrating nuclear genomic analysis are needed to determine whether mtDNA alterations carry prognostic or therapeutic implications.

### Limitations

This retrospective pathology-based study included a relatively small cohort (*n* = 43), and clinical correlations should be considered exploratory, as the study was not powered to detect subtle associations. mtDNA sequencing was feasible only in tumors with high-quality fresh-frozen material (*n* = 21); FFPE-derived DNA was unsuitable for long-range PCR. Nuclear-encoded OXPHOS genes were not analysed due to unavailable funding, and their contribution to respiratory chain defects cannot be excluded. Oncocytic morphology assessment showed limited reproducibility and lacked ultrastructural confirmation. Although immunohistochemistry was standardized, technical variability may have influenced staining intensity.

## Supplementary Information

Below is the link to the electronic supplementary material.

Supplementary Material 1

Supplementary Material 2

Supplementary Material 3

Supplementary Material 4

## Data Availability

Data available on request from the authors.
