# Supplementary material for: Oxidative phosphorylation patterns in pituitary adenoma/neuroendocrine tumors
Source: Pituitary. 2026 Mar 11;29(2):51. doi: 10.1007/s11102-026-01658-w (PMC12979347; doi:10.1007/s11102-026-01658-w)
Supplement: Supplementary file 2 — Supplementary Material 2 [file 11102_2026_1658_MOESM2_ESM.docx]

**Supplementary Methods**

### *mtDNA sequencing*

Long-range PCR amplification

MtDNA was amplified from proteinase K-digested fresh-frozen tumor/adenoma tissue using long-range PCR. For each sample, two PCR reactions were performed using primer sets (Microsynth): primer set A: (forward 5’-CACCAGCCTAACCAGATTTCA-3’; reverse 5’-TGGTACCCAAATCTGCTTCC-3’) and primer set B (forward 5’-GGCTCACATCACCCCATAAA-3’; reverse 5’-CGTGTGGGCTATTTAGGCTTT-3’).

Each PCR reaction contained 1 µl of digested tumor/adenoma sample, 1x LongAmp-Taq (New England Biolabs, NEB), and primers at a final concentration of 0,4 µM. Initial heating to 94°C for 30 seconds, was followed by 40 cycles of 94 °C for 30 seconds, 58 °C for 60 seconds, and 68 °C for 13 minutes. PCR products were purified using the Monarch® PCR & DNA cleanup Kit 5 μg (New England Biolabs, NEB) according to the manufacturer’s instructions.

Long-read mtDNA sequencing

Long-read mtDNA sequencing library was prepared using the Oxford Nanopore Technologies Native Barcoding Kit (SQK-NBD114.24). For each sample, 500 ng of each long-range PCR product were mixed with 0,875 µl NEBNext Ultra II End-prep Reaction Buffer (NEB), 0,75 µl NEBNext Ultra II End-prep Enzyme Mix (NEB) and nuclease-free water to a total volume of 15 µl. The sample was incubated at 20°C for 5 minutes followed by 65°C for 5 minutes. Each sample was mixed with 1x AMPure XP Beads and incubated for 5 minutes on a rotator mixer. The beads were washed twice with 200 µl of 80% ethanol and the sample was eluted in 10 μl nuclease-free water. 7.5 μl of each end-prepped sample were mixed with a unique barcode and 10 μl Blunt/TA Ligase Master Mix (NEB). The reaction was incubated for 20 minutes at RT and stopped by adding 4 μl of 0.25 M EDTA. Barcoded samples were pooled, and 0.4x AMPure XP Beads were added. The sample was incubated for 10 minutes on a rotator mixer and 700 μl of 80% ethanol were used for washing the beads twice. To elute the DNA from the beads, 35 μl nuclease-free water were added and the beads were incubated at 37°C for 10 minutes and every 2 minutes the beads were gently mixed. The beads were pelleted on a magnet and 35 µl of barcoded sequencing library removed. Sequencing adapters were ligated by mixing 30 µl of the barcoded sequencing library with 5 µl Native Adapter, 10 µl NEBNext Quick Ligation Reaction Buffer (NEB) and 5 µl Quick T4 DNA Ligase (NEB) and incubation for 20 minutes at RT. The library was purified by adding 0.4x AMPure XP beads and incubating the reaction for 10 minutes on a rotator mixer at RT. The beads were washed twice by resuspending in 125 μl Long Fragment Buffer, pelleting the beads and removing the supernatant. Finally, the sequencing library was eluted in 35 μl Elution Buffer by incubation for 10 minutes at 37 °C and flicking the beads every 2 minutes to mix.
