# Supplementary material for: Oxidative phosphorylation patterns in pituitary adenoma/neuroendocrine tumors
Source: Pituitary. 2026 Mar 11;29(2):51. doi: 10.1007/s11102-026-01658-w (PMC12979347; doi:10.1007/s11102-026-01658-w)
Supplement: Supplementary file 3 — Supplementary Material 3 [file 11102_2026_1658_MOESM3_ESM.docx]

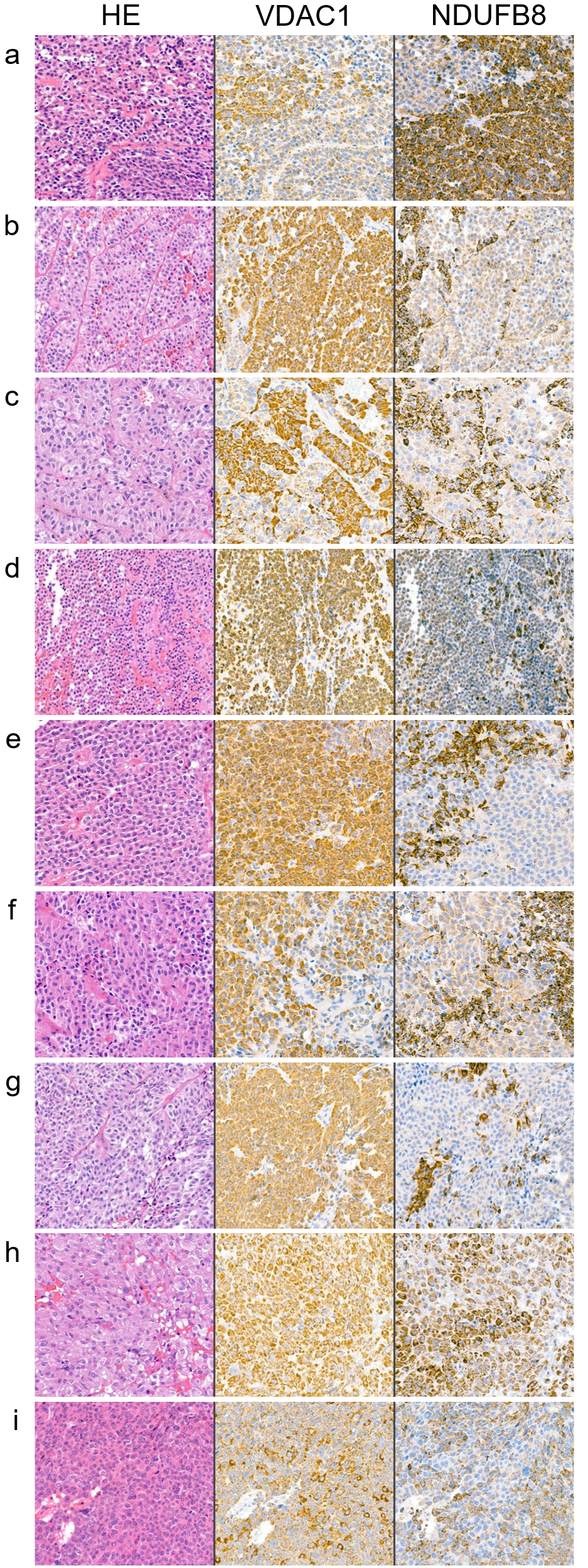
**Supplemental figure 1.** PitNETs/adenomas with detected mtDNA mutations. Representative HE-sections and immunohistochemical stainings for VDAC1 (porin) and NDUFB8 (CI subunit), x20.

SF1-lineage, a-g:

(a) Tumor/adenoma 8, stable genome. The neoplastic tissue mostly showed preserved expression of NDUFB8 with focal deficiency not corresponding with 46 % of mutational load (heteroplasmy), probably due to intratumoral variation in tissue obtained for DNA-isolation and stained tissue. The evident focal CI-deficiency with increased mitochondrial density in VDAC1 is shown.

(b) Tumor/adenoma 9, stable genome. The only tumor/adenoma with mutation affecting MT-TM (mitochondrial tRNA required for ETC complexes I, III, IV, and V). This mutation has been reported to induce a pattern of ETC system multicomplex deficiency [32], as also observed in this tumor/adenoma tissue.

(c) Tumor/adenoma 10 with chromosomal imbalance due to copy-number gains on 7p and 7q.

(d) Tumor/adenoma 30, stable genome.

(e) Tumor/adenoma 32, stable genome.

(f) Tumor/adenoma 34, stable genome.

(g) Tumor/adenoma 51, stable genome.

TPIT-lineage, h-i:

(h) Tumor/adenoma 52 with near-haploid genome with LOH on multiple chromosomes due to whole chromosome loss and expected endoreduplication. (i) Tumor/adenoma 56 with near-haploid genome with LOH on multiple chromosomes due to whole chromosome loss and detected endoreduplication.
