# Supplementary material for: Oxidative phosphorylation patterns in pituitary adenoma/neuroendocrine tumors
Source: Pituitary. 2026 Mar 11;29(2):51. doi: 10.1007/s11102-026-01658-w (PMC12979347; doi:10.1007/s11102-026-01658-w)
Supplement: Supplementary file 4 — Supplementary Material 4 [file 11102_2026_1658_MOESM4_ESM.docx]

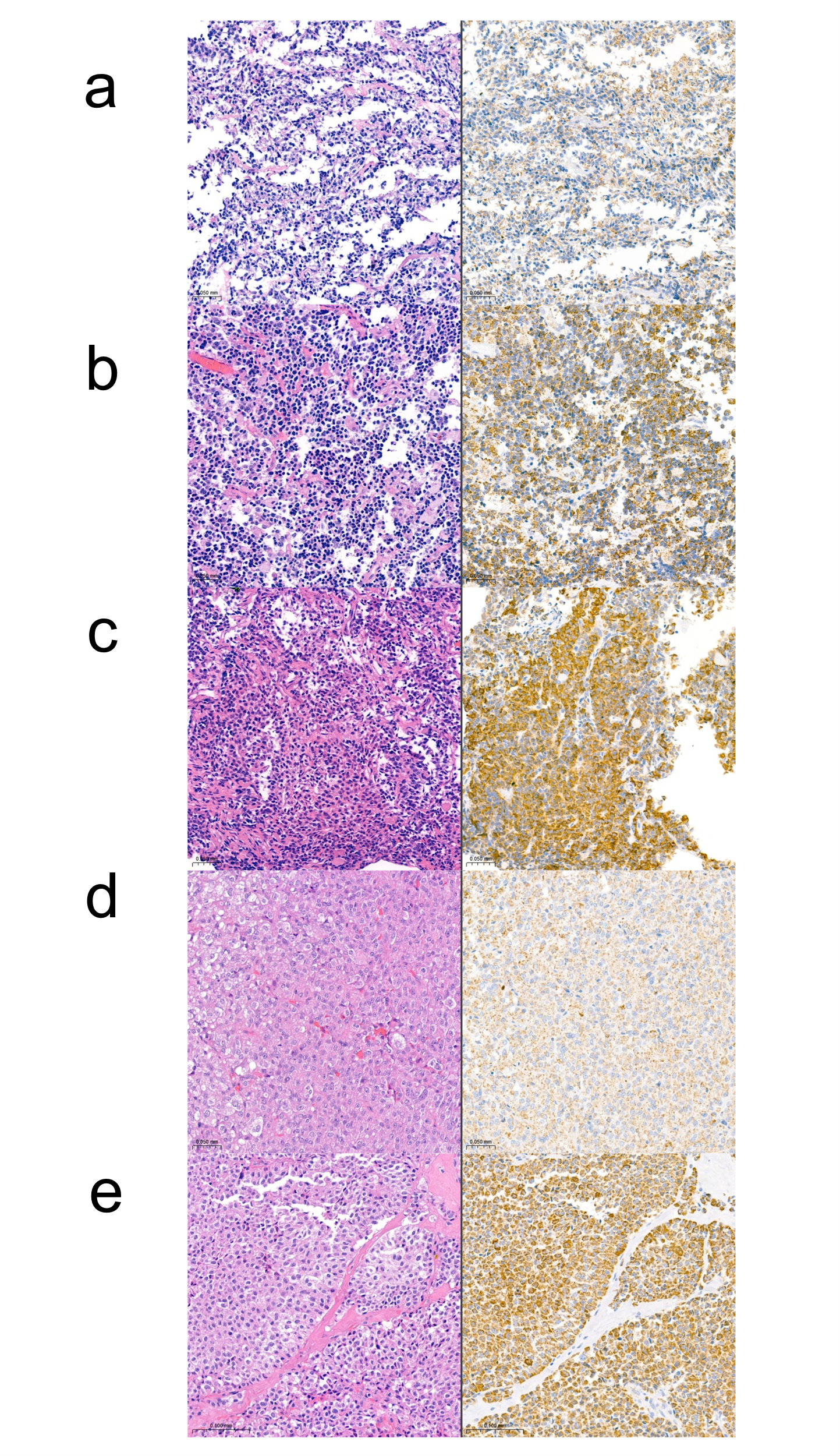
**Supplemental Figure 2.** Representative examples of PitNETs/adenomas with or without oncocytic features and corresponding immunohistochemical VDAC1-expression, x20.

(a) Tumor/adenoma 16, no oncocytic features, VDAC1 intensity 100.

(b) Tumor/adenoma 12, no oncocytic features, VDAC1 intensity 150.

(c) Tumor/adenoma 38, no oncocytic features, overall VDAC1 intensity 130, with areas of reaching staining intensity 250 (shown). No mtDNA mutation detected.

(d) Tumor/adenoma 49 with oncocytic features, VDAC1 intensity varying from 100 (shown) to 150. No mtDNA mutation detected.

(e) Tumor/adenoma 41 with oncocytic features, VDAC1 intensity ranging from 175 to 200 (shown). No mtDNA mutation detected.
